# Identification and Validation of PIK3CA as a Marker Associated with Prognosis and Immune Infiltration in Renal Clear Cell Carcinoma

**DOI:** 10.1155/2021/3632576

**Published:** 2021-07-27

**Authors:** Ya Li, Chong Wang, Yang Gao, Liang Zhou

**Affiliations:** ^1^Department of Clinical Medicine, Dalian Medical University, Dalian 116044, China; ^2^Department of Obstetrics and Gynecology, Shanghai General Hospital, School of Medicine, Shanghai Jiao Tong University, Shanghai, China; ^3^Department of Pathology and Pathophysiology, Hubei Provincial Key Laboratory of Developmentally Originated Disease, School of Basic Medical Sciences, Wuhan University, Wuhan 430071, China; ^4^Department of Urology, Central South University, Changsha 410013, China

## Abstract

**Background:**

Kidney renal clear cell carcinoma (KIRC) is the most prevalent renal malignancy. The therapeutic strategies for advanced KIRC are very few, with only sunitinib being widely approved. Mutations in the PIK3CA gene can affect tumor cell proliferation, metastasis, and patients' survival.

**Methods:**

Bioinformatics analysis was performed to explore the expression and clinical significance of PIK3CA in KIRC. Moreover, qRT-PCR was conducted to verify the result.

**Results:**

Subgroup analyses of KIRC tissue based on gender, tumor grade, and cancer stage indicated downregulation of PIK3CA mRNA expression. The KIRC patients with high PIK3CA expression indicated a better overall survival, progression-free survival, and disease-free survival. A predictive nomogram was constructed and demonstrated that the calibration plots for the 3-year and 5-year OS rates were predicted relatively well compared with an ideal model in the TCGA KIRC cohort. The validation study revealed that downregulation of PIK3CA in KIRC tissues and low PIK3CA expression had a poor overall survival with an AUC of 0.775 in the ROC curve. Moreover, Cox regression analysis revealed that PIK3CA expression and clinical stage were independent factors affecting the prognosis of KIRC patients. PIK3CA expression was found to be significantly associated with the abundance of immune cells and immune biomarker sets. PIK3CA and associated genes were found to be mainly associated with immune response and the JAK-STAT signaling pathway.

**Conclusion:**

We identified PIK3CA as a potential biomarker for prognosis correlated with immune infiltrates in KIRC. Further studies should focus on the functions of PIK3CA in KIRC carcinogenesis.

## 1. Introduction

Kidney renal clear cell carcinoma (KIRC) is the most prevalent malignancy of the kidney and encompasses almost 75% of all kidney cancers [1, 2]. It has been estimated that approximately 66,800 patients were diagnosed with kidney cancer and 13,860 patients died of the disease in 2015 in China [3]. Compared to other types of kidney cancers, KIRC tends to be associated with recurrence, metastasis, radiotherapy, and resistance to chemotherapy [4]. There are very few treatments for advanced KIRC, with sunitinib being the only widely approved drug [5]. Though multidisciplinary synthetic therapy is being used for KIRC, including chemotherapy, immunotherapy, and targeted therapy, the prognosis remains frustrating. The pathogenesis of KIRC is extremely complex, involving multiple genes at multiple steps. Moreover, there are no credible predictive markers for the prognosis and treatment of individual sensitivity or resistance in renal cell carcinoma, though some prognostic factors associated with the survival of RCC patients have been described [6, 7]. Hence, the identification of new drug targets for KIRC by exploring gene networks for changes associated with tumorigenesis and progression has become the focus of numerous studies.

Phosphatidylinositol-4,5-bisphosphate 3-kinase, catalytic subunit alpha (PIK3CA) is an isoform of the PI3K, a significant cell membrane element, and a second messenger involved in cell signaling [8]. Increasing evidence has revealed the presence of common mutations of PIK3CA in various types of cancer, such as gastric cancer, ovarian cancer, colorectal cancer, and lung cancer [8, 9]. These mutations can activate the PI3K/AKT and other downstream signaling pathways, thus affecting tumorigenesis and progression [10]. Moreover, mutant PIK3CA can affect tumor cellular proliferation and invasion, tumor metastasis, and patients' survival [9]. Though some studies have reported a mutation of PIK3CA in RCC, the potential function and clinical significance of PIK3CA remain unknown.

In our current study, we explored the expression, prognosis, and genetic alteration of PIK3CA and its associated enrichment functions and association with immune infiltrates using multidimensional analysis methods across numerous public databases. The data from our study may provide additional information for further studies surrounding the function of PIK3CA in KIRC.

## 2. Methods

### 2.1. TIMER

TIMER (https://www.cistrome.shinyapps.io/timer/) is a comprehensive analysis tool to determine immune-infiltrate levels, clinical association, and therapeutic checkpoint blockade prediction [11]. “Diff Exp” module was utilized to explore differential PIK3CA expression across various tumors. We utilized the “Gene” and “correlation” modules to analyze PIK3CA expression correlation with the abundance of immune cell infiltrates and their gene markers, respectively. Previous studies have described the gene markers associated with several types of immune cells, including CD8+T cells, T cells (general), B cells, monocytes, TAMs, M1 macrophages, M2 macrophages, neutrophils, natural killer (NK) cells, dendritic cells (DCs), T-helper 1 (Th1) cells, T-helper 2 (Th2) cells, follicular helper T (Tfh) cells, T-helper 17 (Th17) cells, Tregs, and exhausted T cells [12–14]. Spearman's correlation test was utilized to analyze the correlation between PIK3CA and immune cell and immune markers. These analyses were performed using the TCGA KIRC dataset (*n* = 538).

### 2.2. UALCAN

UALCAN (http://www.ualcan.path.uab.edu/analysis.html) is a web-based portal that can help accelerate cancer research, including gene expression in cancer subgroups [15]. We submitted PIK3CA to the TCGA analysis module and analyzed PIK3CA expression correlation with clinicopathologic features of KIRC patients at a transcriptional level. The significance of different subgroups was analyzed using Student's *t*-test with *p* value <0.05 as a threshold for statistical significance. These analyses were performed using the TCGA KIRC dataset (*n* = 538).

### 2.3. Oncomine

Oncomine (http://www.oncomine.org) is a versatile systematic platform that comprises oncogene chip databases from TCGA, GEO, and published papers [16]. PIK3CA levels in KIRC patients were compared with those of renal tissues of healthy persons by the Oncomine analysis tool. A *p* value of 0.05 and a fold change of 1.5 were used as the thresholds. Student's *t*-test was then used to evaluate the difference.

### 2.4. The Human Protein Atlas

The Human Protein Atlas (http://www.proteinatlas.org) is a Swedish-based program build to map all the human proteins in cells, tissues, and organs [17]. The protein level of PIK3CA in renal tissue and KIRC tissue was analyzed with the Tissue Atlas and Pathology Atlas of this program.

### 2.5. Prognosis Analysis

The prognosis value of PIK3CA in KIRC was analyzed with the Kaplan–Meier survival analysis. All KIRC samples were divided into a high and low PIK3CA level group with the median value of PIK3CA expression as the cut-off value. A log-rank test was used to evaluate the *p* values and hazard ratio (HR) with a 95% confidence interval (CI). A nomogram was developed based on the results of univariate and multivariate Cox proportional hazards analysis.

### 2.6. cBioPortal

cBioPortal (http://www.cbioportal.org) is an online tool that can perform a systematic analysis of complex cancer genomes and clinical profiles [18]. We utilized cBioPortal to explore, visualize, and analyze PIK3CA genetic alteration and neighboring gene networks. The analysis was performed using a database of 538 TCGA KIRC samples.

### 2.7. DAVID 6.8

DAVID 6.8 (https://www.david.ncifcrf.gov/home.jsp) is an online tool that could perform systematic functional analysis of large gene lists and identify enriched biological functions [19]. In total, 33 related neighboring genes were obtained from cBioPortal. DAVID 6.8 was utilized to perform Gene Ontology (GO) and Kyoto Encyclopedia of Genes and Genomes (KEGG) pathway analysis on these genes.

### 2.8. LinkedOmics

LinkedOmics (https://www.linkedomics.org) is a platform that contains multiomics and clinical data from 11,158 patients from TCGA [20]. In this current study, the TCGA KIRC dataset (538 samples) was used for further analysis using the Pearson correlation test. The LinkFinder module allows accessible exploration of associations between PIK3CA and all additional associated genes. Moreover, PIK3CA and associated genes were submitted to the LinkInterpreter module, which can perform GO, KEGG, kinase targets, miRNA-target enrichment, and transcription factor-target network, thus clarifying the potential functions of PIK3CA and associated genes.

### 2.9. Open Targets

Open Targets (http://www.targetvalidation.org) is an online platform that integrates evidence from genomics, transcriptomics, and scientific literature, which allows for scoring and ranking of target-disease associations for drug target identification [21]. We submitted PIK3CA to this platform and identified the diseases that were associated with PIK3CA.

### 2.10. GeneMANIA

GeneMANIA (http://www.genemania.org) is an online tool that promotes understanding of functional association data of target genes by constructing protein-protein interaction (PPI) network [22]. In order to better understand the function behind these genes associated with the Kinase_MAPK1 network, MIR-200B, MIR-200C, MIR-429, and IPF1 transcription factor network, we submitted these genes to GeneMANIA to construct a PPI network.

### 2.11. GSCALite

GSCALite (http://www.bioinfo.life.hust.edu.cn/web/GSCALite) is an online tool that helps us identify the contribution of a gene to cancer initiation, progress, diagnosis, prognosis, and therapy [23]. GSCALite was used the analyze the cancer pathway activity and drug sensitivity of hub genes (PIK3CA, STRN, C9orf102, REST, and NHLRC2) obtained in LinkedOmics. These analyses were performed using the TCGA KIRC dataset (*n* = 538).

### 2.12. Human Tissues and qRT-PCR

KIRC tissues and pair-normal renal tissues (*n* = 50) were obtained from patients who did not receive any treatment before surgery. All patients provided informed consent. Histological diagnosis and tumor grade were assessed by three experienced pathologists in accordance with 2010 American Joint Committee on Cancer staging system. None of the patients received local or systemic treatment preoperatively.

TRIzol reagent (Vazyme) was used to extract the total RNA of tissue. The synthesis of cDNAs corresponding to the mRNAs of interest depended on PrimeScript RT-polymerase (Vazyme). SYBR-Green Premix (Vazyme) with specific PCR primers. Glyceraldehyde-3-phosphate dehydrogenase was used as an internal control. The 2^−ΔΔ*Ct*^ method was used to calculate fold changes. Primer sequences were as follows: GAPDH, forward: GCACCGTCAAGGCTGAGAAC; reverse: TGGTGAAGACGCCAGTGGA and PIK3CA, forward: AAAGATAACTGAGAAAATGAAAGCTC; reverse: GAAGAAAGCTGACCATGCTGCTATG. The differences in the expression of PIK3CA and the prognosis of PIK3CAin KIRC were evaluated with Student's *t*-test and Kaplan–Meier analysis.

## 3. Results

### 3.1. PIK3CA Level in KIRC

**Figure 1:**
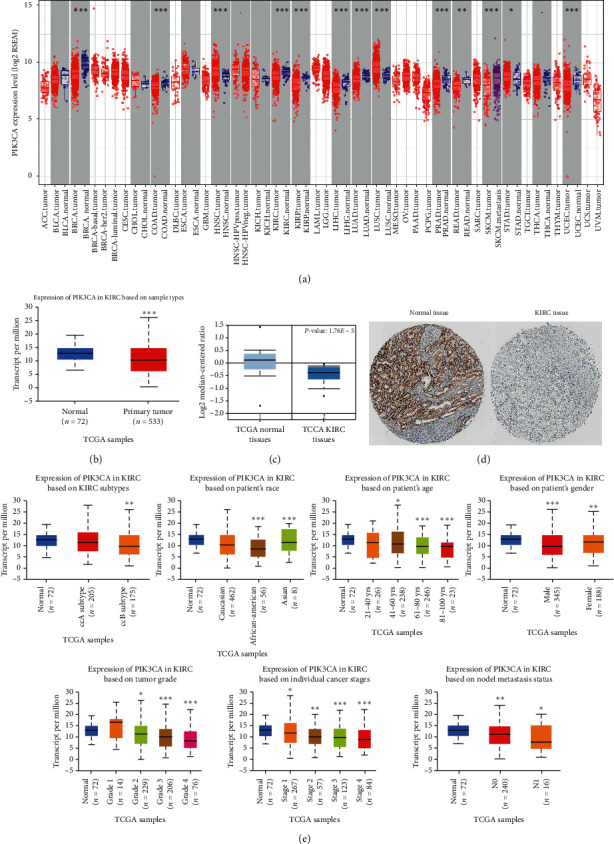
The level of PIK3CA in KIRC. (a) Upregulation or downregulation of PIK3CA in different types of cancers. (b) TCGA dataset showing PIK3CA in KIRC and normal tissue (UALCAN). (c) The mRNA level of PIK3CA in KIRC and normal tissue based on the data from Oncomine. (d) Immunohistochemical staining showing the protein level of PIK3CA in KIRC and normal tissue. (e) The level of PIK3CA in subgroups of patients with KIRC. These analyses were performed using the TCGA KIRC dataset (*n* = 538). ^*∗*^*P* < 0.05, ^∗∗^*P* < 0.01, and ^∗∗∗^*P* < 0.001.

The TIMER database was used to determine differences of PIK3CA expression in tumor and normal tissues across various type of cancers, which demonstrated low PIK3CA mRNA expression in breast cancer, colorectal cancer, KIRC, KIRP, hepatocellular cancer, lung adenocarcinoma, prostate adenocarcinoma, and uterine corpus endometrial carcinoma (Figure 1(a)). Moreover, high PIK3CA mRNA expression was observed in head and neck cancer, breast cancer, colorectal cancer, lung squamous cell carcinoma, and gastric cancer (Figure 1(a)). Furthermore, downregulation of PIK3CA mRNA level was found in KIRC tissues based on UALCAN data (Figure 1(b), *p* < 0.001). According to the result from Oncomine, the mRNA level of PIK3CA was decreased in TCGA KIRC tissues compared with normal tissues with a *p* value of 1.76*E* − 5 (Figure 1(c)). The Human Protein Atlas was used to detect the protein level of PIK3CA in KIRC. As expected, the result suggested a downregulation of PIK3CA protein level in KIRC tissues (Figure 1(d)).

We then evaluated the PIK3CA mRNA expression in subgroup of KIRC patients. Interestingly, the result suggested a downregulation of PIK3CA mRNA levels in KIRC patients compared to normal controls in subgroup analyses based on race, gender, age, KIRC subtypes, tumor grade, cancer stages, and nodal metastasis status (Figure 1(e)). These have indicated a significant role of PIK3CA in the detection of KIRC.

### 3.2. The Prognostic Value of PIK3CA in KIRC

**Figure 2:**
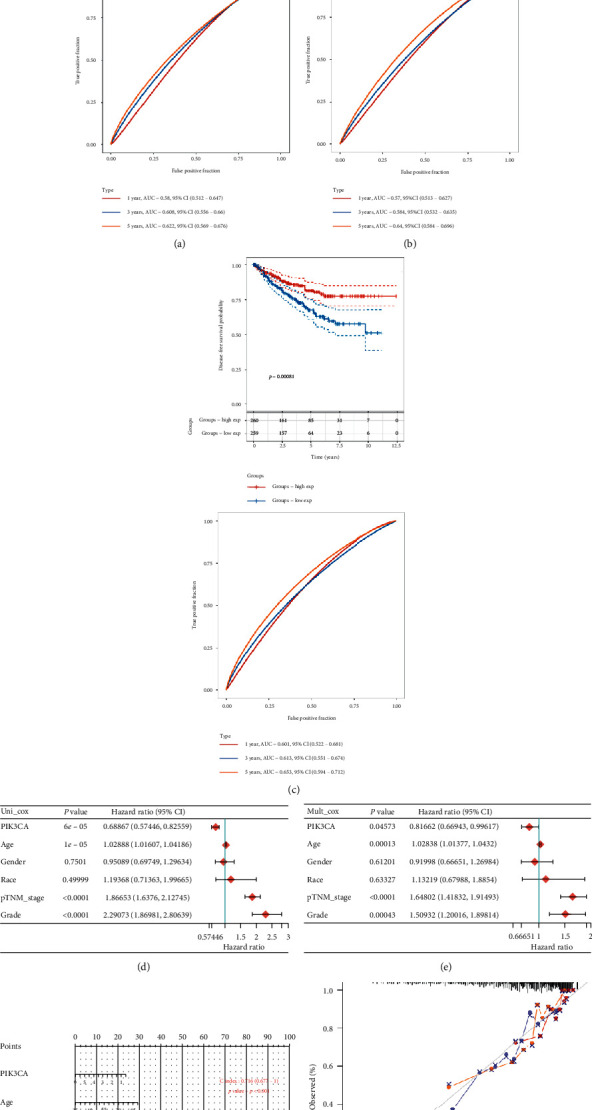
The prognosis analysis of PIK3CA in KIRC. The overall survival curve (a), progression-free survival curve (b), and disease-free survival curve (c) of PIK3CA in KIRC and ROC curve in predicting the prognosis of KIRC patients. (d and e) Hazard ratio and *P* value of constituents involved in univariate and multivariate Cox regression considering clinical parameters and PIK3CA. (f and g) Nomogram to predict the 1 y, 3 y, and 5 y overall survival of KIRC patients. ROC: receiver operating characteristic.

In order to determine the value of PIK3CA in predicting the prognosis of KIRC patients, we divided all KIRC samples into a high and low PIK3CA level group with the median value of PIK3CA expression as the cut-off value. As shown in Figure 2, KIRC patients with low expression of PIK3CA expression had a poor overall survival (OS, *p*=0.00043, Figure 2(a)), progression-free survival (PFS, *p*=0.0083, Figure 2(b)), and disease-free survival (DFS, *p*=0.00081, Figure 2(c)), with a 5-year AUC of 0.622, 0.64 and 0.653, respectively. These results indicated that PIK3CA served as potential prognostic biomarkers in KIRC. The univariate and multivariate analysis revealed that PIK3CA, age, pTNM stage, and tumor grade were independent factors affecting the prognosis of KIRC patients (Figures 2(d) and 2(e)). Moreover, PI3KCA expression distribution in the univariate and multivariable analysis is shown in Supplementary Figure 1. Considering clinicopathologic features and PIK3CA, we constructed a predictive nomogram to predict the 1-year OS, 3-year OS, and 5-year OS rates using the Cox regression algorithm, which demonstrated that the calibration plots for the 3-year and 5-year OS rates were predicted relatively well compared with an ideal model in TCGA KIRC cohort (Figures 2(f) and 2(g)). A Cox proportional hazards model considering age, gender, race, stage and PIK3CA expression was constructed, which further suggested PIK3CA expression as independent factor affecting the prognosis of KIRC patients (Table 1).

### 3.3. Validation of the Expression and Prognostic Value of PIK3CA in KIRC

**Figure 3:**
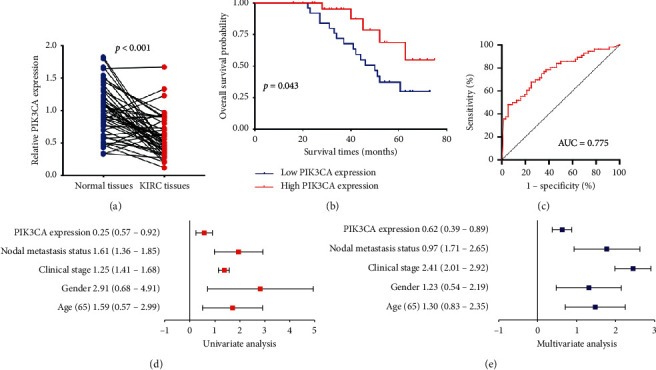
The expression and prognosis value of PIK3CA in KIRC. (a) The relative expression of PIK3CA in KIRC tissues and normal tissues. (b) Survival curve revealed the overall survival of KIRC patients with high/low PIK3CA expression. (c) ROC curve about the prognostic value of PIK3CA in KIRC. (d and e) Univariate and multivariate analysis of PIK3CA and clinical characters in KIRC.

The above results revealed that PIK3CA expression was decreased in KIRC patients and associated with a better prognosis. We then verified the expression and prognostic value of PIK3CA in KIRC by qRT-PCR. All KIRC samples were divided into a high and low PIK3CA level group with the median value of PIK3CA expression as the cutoff value. As expected, the data demonstrated downregulation of PIK3CA in KIRC tissues (*p* < 0.001, Figure 3(a)). Kaplan–Meier curves revealed that KIRC patients with low PIK3CA expression had a poor overall survival (*p*=0.043, Figure 3(b)) with an AUC of 0.775 in the ROC curve (Figure 3(c)), which indicated that PIK3CA has high accuracy in predicting the prognosis of KIRC patients. Moreover, Cox regression analysis revealed that PIK3CA expression and clinical stage were independent factors affecting the prognosis of KIRC patients (Figures 3(e) and 3(f)). These data further confirmed previous results.

### 3.4. The Correlation between PIK3CA Expression and Immune Biomarker Sets in KIRC

**Figure 4:**
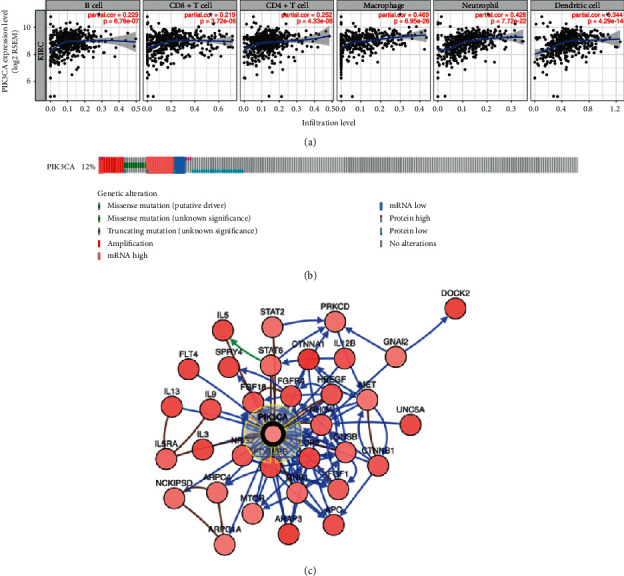
Immune infiltration analysis, genetic alteration, and biological interaction network of PIK3CA in KIRC. (a) Correlation analysis about PIK3CA level and immune cell infiltration in KIRC. (b) PIK3CA genetic alteration in KIRC. (c) Biological interaction network of PIK3CA and the neighbor gene with genetic alteration over 10% in KIRC. These analyses were performed using the TCGA KIRC dataset (*n* = 538).

Previous studies have reported a close relationship between PIK3CA and immune cell or immune response [24, 25]. We also analyzed the relationship between PIK3CA expression, immune cells, and biomarker sets using the KIRC database in TIMER. Results indicated a significant association between PIK3CA levels and abundance of immune cells, including B cells (Cor = 0.229, *P*=6.76*e* − 07), CD8+ T cells (Cor = 0.219, *P*=3.72*e* − 06), CD4+ T cells(Cor = 0.252, *P*=4.33*e* − 08), macrophage (Cor = 0.469, *p*=6.950 − 26), neutrophils (Cor = 0.428, *P*=7.72*e* − 22), and dendritic cells (Cor = 0.344, *P*=4.29*e* − 14) (Figure 4(a)). This was followed by correlation analysis between PIK3CA and immune biomarker sets. As expected, based on the KIRC database of TIMER, there was a significant association between PIK3CA level and most immune marker sets. Our data show the strongest correlation between PIK3CA levels and biomarker datasets of CD8+ T cells, T cells (general), and B cells (Table 2). Surprisingly, for biomarkers of M1 macrophages, positive correlations were found between PIK3CA, INOS, and COX2, while a negative correlation was found in PIK3CA and IRF5. Additionally, PIK3CA levels showed a positive correlation with all biomarkers (CD163, VSIG4, and MS4A4A) of M2 macrophages. We observed that PIK3CA has a negative relationship with all biomarkers of NK and Th1cells, except for KIR3DL3 and TNF-a, respectively. Surprisingly, two biomarkers (FOXP3 and TGFb) of Tregs show a negative correlation, while the other two biomarkers (CCR8 and STAT5B) of Tregs show a positive relationship with PIK3CA in KIRC. For biomarkers of T cell exhaustion, PIK3CA levels were negative correlated with all biomarkers, except for TIM-3. These data demonstrate the contribution of different antigen presentations of tumor-infiltrating immune cells for distinct clinical outcomes of PIK3CA in KIRC.

### 3.5. Genetic Alterations and Neighbor Gene Biological Interaction Network Analysis of PIK3CA in KIRC

**Figure 5:**
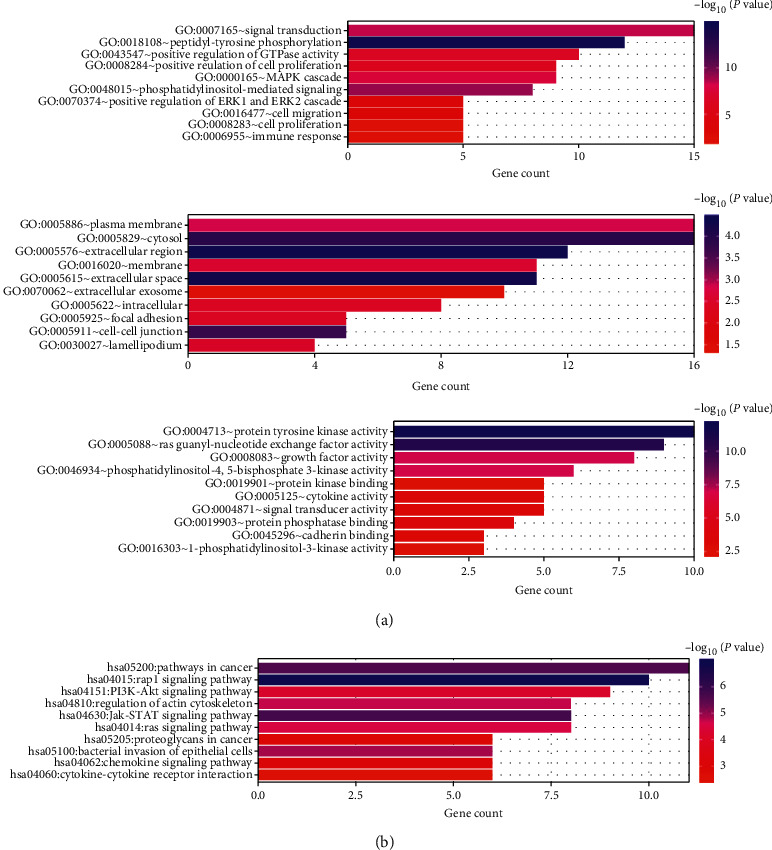
Enrichment analysis of PIK3CA and neighboring genes (DAvid). (a) GO annotation (BP, CC, MF) analysis. (b) KEGG pathway analysis. GO: Gene Ontology; BP: biological processes; CC: cellular components; MF: molecular functions.

Due to the significance of PIK3CA in KIRC, we next sought to explore any PIK3CA genetic alterations. Among each of the 537 TCGA KIRC patients, 64 (12%) patients had a PIK3CA genetic alteration, including 9 (1.67%) with a mutation, 9 (1.67%) with an amplification, 11 (2.04%) with mRNA upregulation, 4 with mRNA downregulation, 3 (0.56%) with protein upregulation, 23 (4.28%) with protein downregulation, and 5 (0.93%) with multiple alterations (Figure 4(b)). We also constructed a PIK3CA associated gene network, which revealed 33 neighboring genes (APC, APAR3, ARPC1A, ARPC4, CTNNA1, CTNNB1, DOCK2, FGF1, FGF18, FGFR4, FLT4, GNAI2, GNB1, GUSB, HBEGF, IL12B, IL13, IL3, IL5, IL5RA, IL9, LCP2, MET, MTOR, NCKIPSD, NRG2, PDGFRB, PRKCD, RHOA, SPRY4, STAT2, STAT6, and UNC5A) that had genetic alterations in more than 10% of patients (Figure 4(c), Table 3). Among these neighboring genes, CTNNA1 (20.8%), APAR3 (20.8%), and DOCK2 (20.8%) ranked among the three most frequent alterations (Table 3). Moreover, we performed GO and KEGG pathway analyses of these neighboring genes. Results indicated that neighboring genes were mainly associated with signal transduction, cell migration and proliferation, immune response, cytokine activity, and kinase binding and activity in GO functions (Figure 5(a)). These neighboring genes were mainly associated with cancer-related pathways, Rap1 signaling pathway, P13K-Akt signaling pathway, JAK-STAT signaling pathway, and chemokine signaling pathway (Figure 5(b)).

### 3.6. GO and KEGG Pathway Analyses of Coexpression Genes Correlated with PIK3CA in KIRC

**Figure 6:**
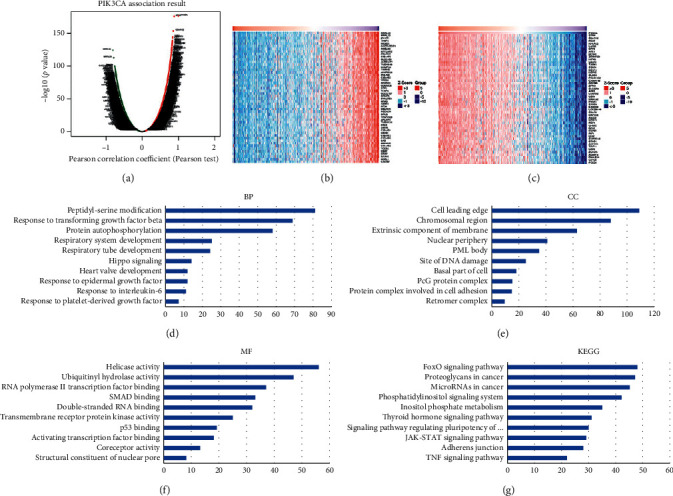
Correlated genes, GO and KEGG pathways analysis of PIK3CA in KIRC. (a) PIK3CA association result in KIRC. (b and c) Heat maps of the most significant 50 genes positively and negatively correlated with PIK3CA in KIRC. (d) BP analysis. (e) CC analysis. (f) MF analysis. (g) KEGG pathway analysis.

**Figure 7:**
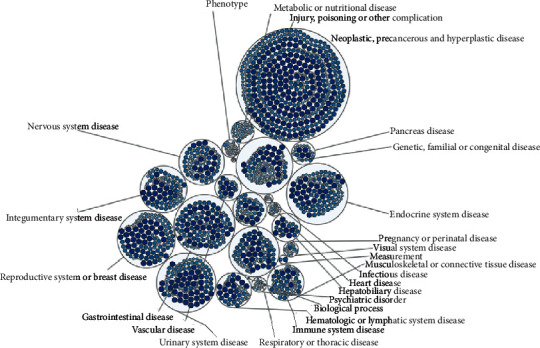
Bubble map revealing the diseases associated with PIK3CA.

We submitted PIK3CA to LinkedOmics and analyzed the mRNA expression of 533 TCGA KIRC patients. The correlation analysis revealed 6680 genes (dark red dots) that were positively correlated with PIK3CA, while 7687 genes (dark green dots) negatively correlated with PIK3CA, with a false discovery rate <0.05. Figures 6(b) and 6(c) show the 50 genes that are most positively and negatively associated with PIK3CA (Figure 6(a)). We selected the most significant four genes that were positively associated with PIK3CA, including STRN (cor = 0.8826, *p*=3.4*e* − 176), c9orf102 (cor = 0.8559, *p*=3.581*e* − 154), EST (cor = 0.8436, *p*=1.62*e* − 145), and NHLRC2 (cor = 0.8421, *p*=1.726*e* − 144) as the hub genes for further study (Supplementary Figure 2). Moreover, we performed GO and KEGG pathway analyses. GO results suggested that the PIK3CA-associated genes are involved in peptidyl-serine modification, hippo signaling, chromosomal region, DNA damage, double-stranded RNA binding, and P53 binding (Figures 6(d)–6(f)). Results from the KEGG pathway suggest the involvement of PIK3CA-associated genes in the FoxO signaling pathway, proteoglycans in cancer, microRNAs in cancer, and the JAK-STAT signaling pathway (Figure 6(g)). Moreover, the results of Open Targets also suggest that PIK3CA was associated with immune system disease and urinary system disease (Figure 7).

### 3.7. PIK3CA Networks of Kinase and miRNA Targets in KIRC

**Figure 8:**
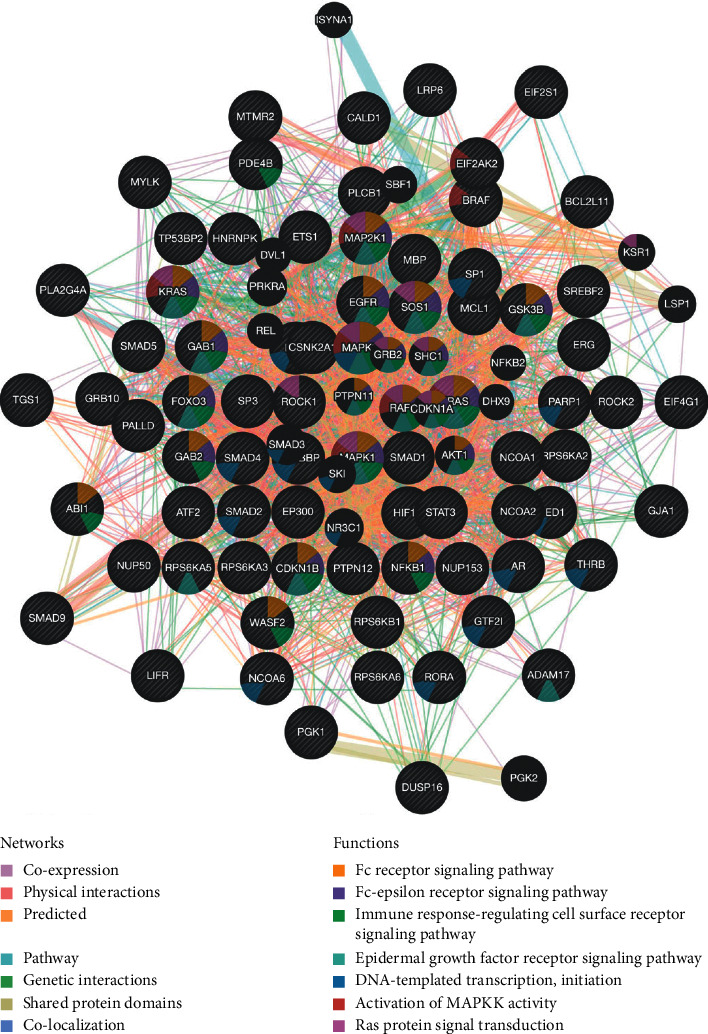
PPI network of MAPK1 kinase target networks. PPI network and functional analysis of the gene sets of MAPK1 kinase target networks. The different colors for the network nodes indicate the biological functions of the set of enrichment genes.

Due to the significant clinical role of PIK3CA in KIRC, we explored kinase, miRNA o targets of PIK3CA using LinkedOmics. For kinase targets of PIK3CA in KIRC, the top 5 most significant genes included MAPK1, ATM, CSNK1D, FER, and EGFR (Table 4). We also constructed a PPI network based on the genes correlated with kinases MAPK1 target network, which suggested enrichment in the Fc receptor signaling pathway, immune response, DNA-templated transcription and initiation, and activation of MAPKK activity (Figure 8, Supplementary Table 1). Table 4 also shows the top 5 most significant miRNA targets, including MIR-200B, MIR-200C and MIR-429 (CAGTATT); MIR-181A, MIR-181B, MIR-181C, and MIR-181D (TGAATGT); MIR-130A, MIR-301, and MIR-130B (TTGCACT); MIR-519C, MIR-519B, and MIR-519A (TGCACTT); MIR-141 and MIR-200A (CAGTGTT). The PPI network based on the target network of MIR-200B, MIR-200C, and MIR-429 (Supplementary Figure 3, Supplementary Table 2) revealed their functions in small conjugating protein ligase activity, transcription coactivator activity, nuclear matrix, and periphery.

### 3.8. Cancer Pathway Activity and Drug Sensitivity Analysis of Hub Genes in KIRC

**Figure 9:**
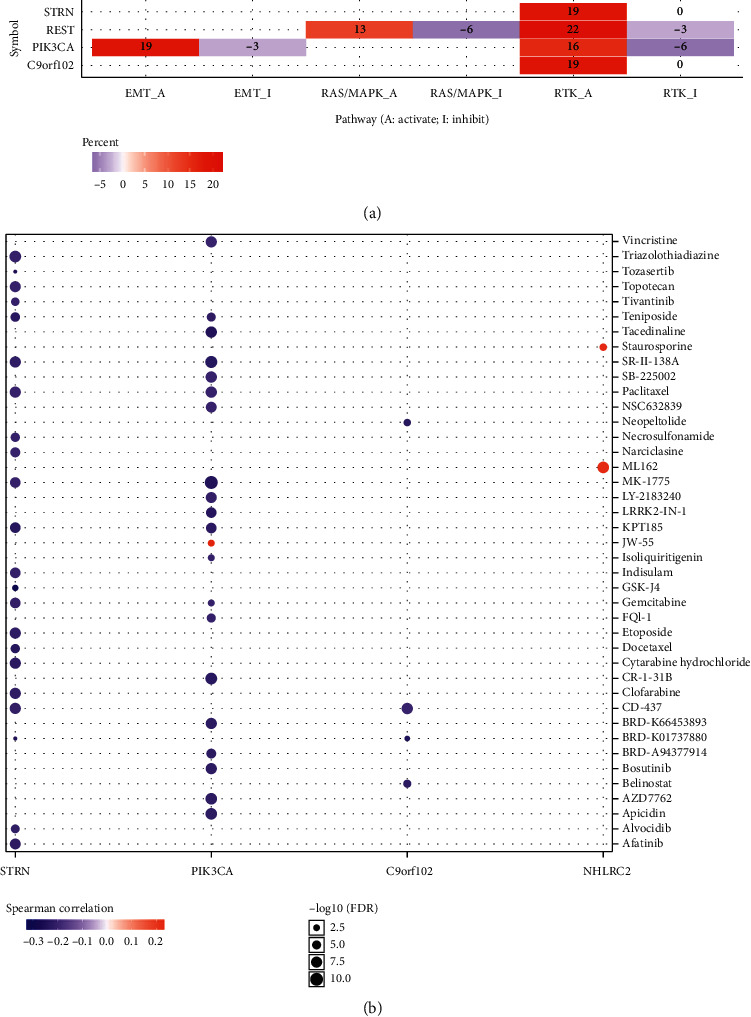
Cancer pathway activity and drug sensitivity analysis of hub genes in KIRC. (a) The activation and inhibition of hub genes (PIK3CA, STRN, C9orf102, REST, and NHLRC2) in cancer-related signaling pathways in KIRC. (b) The correlation between the level of hub genes drug (PIK3CA, STRN, C9orf102, REST, and NHLRC2) and drug sensitivity in KIRC. These analyses were performed using the TCGA KIRC dataset (*n* = 538).

The hub genes (PIK3CA, STRN, C9orf102, REST, and NHLRC2) obtained from LinkedOmics were submitted to GSCALite for cancer pathway activity and drug sensitivity analysis. We found that these hub genes were mainly responsible for the activation of EMT pathways, RAS/MAPK pathways, and RTK pathways, among other famous cancer-related pathways (Figure 9(a)). The results of the drug sensitivity analysis are shown in Figure 9(b). The small molecules or drugs from the Therapeutics Response Portal were analyzed, which revealed that low PIK3CA level is resistant to 19 small molecules or drugs. Moreover, low STRN is resistant to 22 small molecules or drugs. Thus, STRN and PIK3CA may be promising biomarkers for drug screening.

## 4. Discussion

PI3K signaling pathway is crucial for tumor cell migration, growth, metastasis, and survival [26]. Several lines of evidence have revealed extensive PIK3CA gene mutations across various types of tumors [27]. PIK3CA is involved in tumor progression and multidrug resistance, thus affecting overall survival. While some studies have reported mutation of PIK3CA in RCC, the potential functions and clinical significance of PIK3CA remain unclear. Thus, we carried out our study to identify the significance of PIK3CA in KIRC.

We initially explored the expression of PIK3CA KIRC, which revealed that the mRNA and protein levels of PIK3CA were significantly lower in tumor tissues than in normal tissues. Moreover, for subgroup analyses based on race, gender, age, KIRC subtypes, tumor grade, cancer stages, and nodal metastasis status, PIK3CA mRNA expression was also significantly lower in tumor tissues than in normal tissues. Interestingly, high PIK3CA mRNA level was also observed in breast cancer, colorectal cancer, head and neck cancer, lung squamous cell carcinoma, and gastric cancer. These results were in agreement with the fact that the expressions of the same gene in different tumors were very different. In KIRC, the mRNA level of PIK3CA was decreased. That is probably because tumor tissues could inhibit the level of PIK3CA or PIK3CA served as a tumor suppressor in KIRC. The prognostic analysis revealed that the KIRC patients with high PIK3CA levels had improved overall survival, which suggested that PIK3CA may function as a potential biomarker for detection and prognostic prediction in KIRC. In cervical cancer, PIK3CA was a prognostic biomarker and associated with poor overall survival [28]. Moreover, PIK3CA in residual disease after neoadjuvant chemotherapy is associated with poor survival in breast cancer, suggesting PIK3CA as a prognostic biomarker in breast cancer [29]. Another study revealed that PIK3CA-mutated HR+/Her2− breast cancer patients had a poor outcome and resistance to chemotherapy [30].

Previous studies had reported a close relationship between PIK3CA and immune cells or immune response in cancers, including lung and colorectal cancer [31, 32]. In this current study, we analyzed the correlation between PIK3CA expression and immune cells and biomarker sets in KIRC. As expected, there was a significant association between PIK3CA expression and the abundance of immune cells. Dendritic cells (DCs), the important parts of a tumor microenvironment, can infiltrate into tumors, thus activating immune response and recruiting disease-fighting immune effector cells and pathways, making DCs a potential therapeutic target for cancer immune therapy [33]. CD8+ T cells and CD4+ T cells were crucial for tumor progression [34]. These endothelial cells in angiogenesis in a tumor microenvironment played a role in hypoxia, which is a key factor for angiogenesis. Moreover, it would be better to clarify the association among endothelial cells, PIK3CA, and angiogenesis. We also observed that most immune cell biomarkers positively or negatively correlated with PIK3CA. Accumulating evidence has revealed the functions of these immune biomarkers in the tumor microenvironment and immune response. Blockade of PD-1, CTLA-4, and TIM-3 can enhance cetuximab-based cancer immunotherapy to reverse CD8+ tumor-infiltrating lymphocytes dysfunction, thus affecting the prognosis of cancer patients [35]. Infiltration of CD8+ T cells in RCC was associated with a favorable prognosis [36]. Moreover, macrophages were also associated with a favorable outcome in RCC. In our study, we found that levels of PIK3CA positively correlate with CD8+ T cells and macrophages. Therefore, PIK3CA may regulate tumor cellular progression and immune infiltration by uniting CD8+ T cells and macrophages, thus affecting KIRC patients' survival.

Another important finding of our study was that PIK3CA and its associated genes were mainly involved in signal transduction, cell migration and proliferation, immune response, cytokine activity, P13K-Akt signaling pathway, and JAK-STAT signaling pathway. In fact, the processes and signaling pathways were associated with tumorigenesis and cancer progression. Abrogation of PIK3CA could inhibit tumor cell proliferation, migration, and invasion in glioblastoma [37]. Previous studies have suggested that aberrant JAK/STAT signaling is crucial for tumor progression and metastatic development [38]. Many members of JAK/STAT signaling were found to be associated with a prognosis of KIRC. JAK3 in KIRC was a marker for predicting poor survival in KIRC [1]. STAT2/4/5B was also suggested to be prognostic biomarkers in KIRC [39]. Therefore, PIK3CA may regulate tumorigenesis and progression via JAK/STAT signaling, thus affecting the prognosis of KIRC.

In order to better clarify the mechanism of PIK3CA in KIRC, we identified several kinases, miRNA, and transcription factor-target networks of PIK3CA in KIRC. Interestingly, the identified kinases, such as MAPK1 and EGFR, were associated with tumor proliferation, invasion, apoptosis, and angiopoiesis. In breast cancer, PIK3CA exon-specific mutations can enhance MAPK1/3 phosphorylation, contributing to a favorable prognosis [40]. In endometrial cancer, miR-143 suppressed tumor cell proliferation and metastasis by regulating MAPK1 [41]. A previous study suggested the involvement of EGFR in the pathogenesis and progression of cancers [42]. Interestingly, EGFR was found to be associated with a good prognosis in RCC [43]. Actually, increasing studies focus on the molecular mechanisms of neoplastic transformation and progression, which have been performed to identify novel drugs, including EGFR tyrosine kinases inhibitors in cancer treatment [44]. Therefore, PIK3CA can affect tumor proliferation, invasion, apoptosis, and angiopoiesis in KIRC by regulating MAPK1 and EGFR.

We also identified several miRNA targets of PIK3CA in KIRC, including miR-200B, miR-200C, and miR-141. Interestingly, a previous study revealed that miR-141, miR-200B, and miR-200C are prognostic biomarkers of KIRC and associated with better prognosis [45]. Moreover, miR-200B was downregulated and inhibited metastasis in KIRC [46]. A downregulation of miR-200c-3p in RCC tissues suppresses tumor cell proliferation and invasion and induces apoptosis [47]. Another study suggested that miR-141 is a biomarker in KIRC and inhibits tumor cell proliferation and metastasis [48]. Therefore, miR-200B, miR-200C, and miR-141 may regulate cellular progression in KIRC and affect patients' prognosis by targeting PIK3CA. Further studies should be performed to validate these findings.

Moreover, we also conducted cancer pathway activity and drug sensitivity analysis of hub genes (PIK3CA, STRN, C9orf102, REST, and NHLRC2) obtained from LinkedOmics. Our findings indicated that these hub genes were mainly responsible for activating EMT pathways, RAS/MAPK pathways, and RTK pathways, among these other famous cancer-related pathways, which suggested that PIK3CA can exert functions in KIRC via these signaling pathways. The results from drug sensitivity analysis revealed that low PIK3CA is resistant to 19 small molecules or drugs, demonstrating that PIK3CA is a promising biomarker for drug screening.

Indeed, a decision-making strategy based on clinical-histopathological criteria had been developed for renal cell carcinoma, and we could predict the prognosis of patients based on the subtype so that different drugs or treatment regimens can be selected for therapy, reducing treatment-related complications [6]. We should identify more reliable predictive biomarkers of treatment individual sensitivity or resistance, thus prompting precise and individualized treatment.

There is no doubt that some limitations were found in the current study. First, the main limitation of the paper is the methodology used for the analysis. Moreover, subjective cluster annotation, which was not well-defined in the current study, can also be dangerous. Moreover, it would be better if our results were verified by another database, such as GEO. Finally, it would be better to perform prognostic analysis based on different clinicopathological features. Further studies should focus on the molecular biological functions and potential mechanisms of PIK3CA in KIRC.

## 5. Conclusions

Overall, we identified PIK3CA as a potential prognostic biomarker that is associated with immune cell infiltration, providing additional data for further study of the functions of PIK3CA in KIRC carcinogenesis. Further study should be performed to verify our results.

## Figures and Tables

**Table 1:** A Cox proportional hazards model considering age, gender, race, stage, and PIK3CA expression in KIRC.

—	Coef	HR	95% CI_l	95% CI_u	*p* value	Sig
Age	0.030	1.031	1.016	1.046	≤0.001	^∗∗∗^
Gender: male	−0.129	0.879	0.636	1.216	0.436	—
Race: Black	0.307	1.360	0.173	10.704	0.770	—
Race: White	0.461	1.585	0.218	11.545	0.649	—
Stage 2	0.201	1.223	0.654	2.289	0.529	—
Stage 3	0.879	2.408	1.589	3.649	0.000	^∗∗∗^
Stage 4	1.898	6.674	4.526	9.841	0.000	^∗∗∗^
PIK3CA	−0.288	0.750	0.624	0.901	0.002	^∗∗^

*R* square = 0.223. Score (log-rank) test *p*=3.79*e* − 30, ^∗∗^*p* < 0.01; ^∗∗∗^*P* < 0.001.

**Table 2:** Correlation analysis between PIK3CA and related genes and markers of immune cells in KIRC (TIMER).

Description	Gene markers	KIRC			
		None		Purity	
		Cor	*P* value	Cor	*P* value
CD8+ T cell	CD8A	−0.155	^∗∗∗^	−0.158	^∗∗∗^
	CD8B	−0.208	^∗∗∗^	−0.215	^∗∗∗^

T cell (general)	CD3D	−0.267	^∗∗∗^	−0.28	^∗∗∗^
	CD3E	−0.228	^∗∗∗^	−0.236	^∗∗∗^
	CD2	−0.179	^∗∗∗^	−0.183	^∗∗∗^

B cell	CD19	−0.16	^∗∗∗^	−0.155	^∗∗∗^
	CD79A	−0.155	^∗∗∗^	−0.16	^∗∗∗^

Monocyte	CD86	0.057	0.19	0.076	0.101
	CD115 (CSF1R)	0.141	^∗∗^	0.145	^∗∗^

TAM	CCL2	−0.141	^∗∗^	−0.097	^*∗*^
	CD68	−0.055	0.207	−0.047	0.31
	IL10	0.172	^∗∗∗^	0.171	^∗∗∗^

M1 macrophage	INOS (NOS2)	0.236	^∗∗∗^	0.224	^∗∗∗^
	IRF5	−0.414	^∗∗∗^	−0.389	^∗∗∗^
	COX2 (PTGS2)	0.188	^∗∗∗^	0.212	^∗∗∗^

M2 macrophage	CD163	0.376	^∗∗∗^	0.374	^∗∗∗^
	VSIG4	0.144	^∗∗∗^	0.131	^∗∗^
	MS4A4A	0.207	^∗∗∗^	0.22	^∗∗∗^

Neutrophils	CD66b (CEACAM8)	0.069	0.11	0.062	0.184
	CD11b (ITGAM)	0.135	^∗∗^	0.144	^∗∗^
	CCR7	−0.039	0.374	−0.033	0.484

Natural killer cell	KIR2DL1	−0.091	^*∗*^	−0.117	^*∗*^
	KIR2DL3	−0.106	^*∗*^	−0.131	^∗∗^
	KIR2DL4	−0.247	^∗∗∗^	−0.253	^∗∗∗^
	KIR3DL1	−0.111	^∗∗^	−0.131	^∗∗^
	KIR3DL2	−0.171	^∗∗∗^	−0.163	^∗∗∗^
	KIR3DL3	−0.024	0.58	−0.001	0.987
	KIR2DS4	−0.107	^*∗*^	−0.119	^*∗*^

Dendritic cell	HLA-DPB1	−0.106	^*∗*^	−0.114	^*∗*^
	HLA-DQB1	−0.134	^∗∗^	−0.136	^∗∗^
	HLA-DRA	0.015	0.726	0.016	0.739
	HLA-DPA1	0.011	0.805	0.022	0.645
	BDCA-1 (CD1C)	0.08	0.0662	0.082	0.0786
	BDCA-4 (NRP1)	0.458	^∗∗∗^	0.458	^∗∗∗^
	CD11c (ITGAX)	−0.187	^∗∗∗^	−0.178	^∗∗∗^

Th1	T-bet (TBX21)	−0.186	^∗∗∗^	−0.182	^∗∗∗^
	STAT4	−0.206	^∗∗∗^	−0.202	^∗∗∗^
	STAT1	0.234	^∗∗∗^	0.246	^∗∗∗^
	IFN-g (IFNG)	−0.151	^∗∗∗^	−0.16	^∗∗∗^
	TNF-a (TNF)	−0.075	0.0853	−0.062	0.184

Th2	GATA3	−0.149	^∗∗∗^	−0.141	^∗∗^
	STAT6	−0.187	^∗∗∗^	−0.191	^∗∗∗^
	STAT5A	−0.096	^*∗*^	−0.091	0.0505
	IL13	−0.221	^∗∗∗^	−0.162	^∗∗∗^

Tfh	BCL6	−0.042	0.338	−0.038	0.42
	IL21	0.075	0.0828	0.07	0.132

Th17	STAT3	0.456	^∗∗∗^	0.458	^∗∗∗^
	IL17A	−0.026	0.548	0.012	0.801

Treg	FOXP3	−0.183	^∗∗∗^	−0.17	^∗∗∗^
	CCR8	0.098	^*∗*^	0.131	^∗∗^
	STAT5B	0.389	^∗∗∗^	0.404	^∗∗∗^
	TGFb (TGFB1)	−0.101	^*∗*^	−0.119	^*∗*^

T cell exhaustion	PD-1 (PDCD1)	−0.276	^∗∗∗^	−0.269	^∗∗∗^
	CTLA4	−0.155	^∗∗∗^	−0.146	^∗∗^
	LAG3	−0.288	^∗∗∗^	−0.29	^∗∗∗^
	TIM-3 (HAVCR2)	−0.004	0.93	−0.005	0.915
	GZMB	−0.299	^∗∗∗^	−0.326	^∗∗∗^

These analyses were performed using the TCGA KIRC dataset (*n* = 538). ^*∗*^*p* < 0.05; ^∗∗^*p* < 0.01; ^∗∗∗^*P* < 0.001.

**Table 3:** The type and frequency of PIK3CA neighbor gene alterations in KIRC (cBioPortal).

Gene symbol	Amplification	Homozygous deletion	Upregulation	Downregulation	Mutation	Total alteration
APC	9.3	0.2	7.2	0.9	1.3	10.4
APAR3	13	0	4.3	0	4.6	21
ARPC1A	0.7	0	10	0.4	0	10.6
ARPC4	0	10.6	0.7	0	0	11.3
CTNNA1	13.4	0.2	11	1.7	0.9	24.5
CTNNB1	0.2	10.4	3.2	2.2	0.2	15.6
DOCK2	14.7	0	5.2	0	1.3	20.1
FGF1	13.8	0	0.9	0	0	14.5
FGF18	14.9	0	0.9	0	0	15.6
FGFR4	15.4	0	2.2	0	0.6	17.7
FLT4	15.1	0	4.1	1.5	0	19.1
GNAI2	0	10.4	0.6	0	0.2	11.2
GNB1	0	0.2	1.3	10.2	0.4	12.1
GUSB	0.2	0	11	0.7	0.7	12.5
HBEGF	0.2	0	11	0.7	0.7	16.0
IL12B	14.1	0	2.6	0	0	16.5
IL13	13.2	0	5.8	0	0	18
IL3	13.2	0	6.3	0	0.2	19
IL5	13.2	0	6.7	0	0	18.4
IL5RA	0	10.2	3.2	0	0	13.4
IL9	13.4	0	3.9	0	0	16.9
LCP2	14.9	0	3.7	0	0.2	18.2
MET	10.6	0	9.3	0.2	1.2	10.6
MTOR	0	0.2	3.0	1.5	5.9	10.4
NCKIPSD	0	10.4	0.7	0	0.7	11.9
NRG2	13.8	0	1.5	0	0.4	15.6
PDGFRB	13.8	0	5.4	0	0.2	18.6
PRKCD	0	9.3	1.3	0	1.3	11.3
RHOA	0	10.4	0.4	4.1	0	14.5
SPRY4	13.8	0	3.9	0	1.9	18.8
STAT2	0	0	8.4	0	3.2	11.3
STAT6	0	0	7.6	3	2.2	12.5
UNC5A	15.1	0	2.8	0	0.4	18

**Table 4:** The kinase and miRNA-target networks of PIK3CA in KIRC (LinkedOmics).

Enriched category	Geneset	LeadingEdgeNum	*P* value
Kinase target	Kinase_MAPK1	74	≤0.001
	Kinase_ATM	123	≤0.001
	Kinase_CSNK1D	8	≤0.001
	Kinase_FER	6	≤0.001
	Kinase_EGFR	17	≤0.001

miRNA target	CAGTATT, MIR-200B, MIR-200C, MIR-429	198	≤0.001
	TGAATGT, MIR-181A, MIR-181B, MIR-181C, MIR-181D	206	≤0.001
	TTGCACT, MIR-130A, MIR-301, MIR-130B	151	≤0.001
	TGCACTT, MIR-519C, MIR-519B, MIR-519A	185	≤0.001
	CAGTGTT, MIR-141, MIR-200A	124	≤0.001

## Data Availability

The analyzed datasets generated during the study are available from the corresponding author on reasonable request.

## Abbreviations

KIRC: Clear cell renal cell carcinoma
PIK3CA: Phosphatidylinositol-4,5-bisphosphate 3-kinase, catalytic subunit alpha
GO: Gene Ontology
KEGG: Kyoto Encyclopedia of Genes and Genomes
BP: Biological processes
CC: Cellular components
MF: Molecular functions
PPI: Protein-protein interaction.

## Supplementary Materials

Supplementary Materials Supplementary Figure 1: the distribution of PI3KCA expression in univariate and multivariable analysis. Supplementary Figure 2: correlation analysis of PIK3CA expression and the expression of STRN, C9orf102, REST, and NHLRC2 (LinkedOmics). Supplementary Figure 3. PPI network of MIR-200B, MIR-200C, and MIR-429 target networks (GeneMANIA). PPI network and functional analysis about the gene sets of MIR-200B, MIR-200C, and MIR-429 target networks. The different colors for the network nodes indicate the biological functions of the set of enrichment genes. Supplementary Table 1: the LeadingEdgeGene of MAPK1-kinase target network (LinkedOmics). Supplementary Table 2: the LeadingEdgeGene of MIR-302C target network (LinkedOmics).
