# Supplementary material for: Identification and Validation of PIK3CA as a Marker Associated with Prognosis and Immune Infiltration in Renal Clear Cell Carcinoma
Source: J Oncol. 2021 Jul 27;2021:3632576. doi: 10.1155/2021/3632576 (PMC8337125; doi:10.1155/2021/3632576)
Supplement: Supplementary Materials — Supplementary Figure 1: the distribution of PI3KCA expression in univariate and multivariable analysis. Supplementary Figure 2: correlation analysis of PIK3CA expression and the expression of STRN, C9orf102, REST, and NHLRC2 (LinkedOmics). Supplementary Figure 3. PPI network of MIR-200B, MIR-200C, and MIR-429 target networks (GeneMANIA). PPI network and functional analysis about the gene sets of MIR-200B, MIR-200C, and MIR-429 target networks. The different colors for the network nodes indicate the biological functions of the set of enrichment genes. Supplementary Table 1: the LeadingEdgeGene of MAPK1-kinase target network (LinkedOmics). Supplementary Table 2: the LeadingEdgeGene of MIR-302C target network (LinkedOmics). [file 3632576.f1.zip › 3632576.f1/Supplementary tables.docx]

**Supplementary Table 1. The LeadingEdgeGene of MAPK1-Kinase target network (LinkedOmics).**

| **Description** | **Leading**  **EdgeNum** | **P-value** | **LeadingEdgeGene** |
| --- | --- | --- | --- |
| Kinase_  MAPK1 | 74 | 0 | ABI1, ADAM17, AR, ATF2, BCL2L11, BRAF, CALD1, CDKN1B, CREBBP, CSNK2A1, DUSP16, EGFR, EIF2AK2, EIF2S1, EIF4G1, EP300, ERG, ETS1, FOXO3, GAB1, GAB2, GJA1, GRB10, GSK3B, GTF2I, HIF1A, HNRNPK, KRAS, LIFR, LRP6, MAP2K1, MAPK1, MBP, MCL1, MED1, MTMR2 MYLK, NCOA1, NCOA2, NCOA6, NFKB1, NRAS NUP153, NUP50, PALLD, PARP1, PDE4B, PGK1, PGR, PLA2G4A, PLCB1, PTPN12, ROCK1, ROCK2, RORA, RPS6KA2, RPS6KA3, RPS6KA5, RPS6KA6, RPS6KB1, SMAD1, SMAD2, SMAD4, SMAD5, SMAD9, SOS1, SP1, SP3, SREBF2, STAT3, TGS1, THRB, TP53BP2, WASF2 |

**Supplementary Table 2. The LeadingEdgeGene of MIR-302C target network (LinkedOmics).**

| **Description** | **Leading**  **EdgeNum** | **P-value** | **LeadingEdgeGene** |
| --- | --- | --- | --- |
| CAGTATT, MIR-200B, MIR-200C, MIR-429 | 198 | 0 | MED13, PPP4R2, NUP153, NCOA2, APPL1, PIKFYVE, FBXW11, CTDSPL2, CHD9, ATRX, HIPK1, AKAP2, PDS5B, RPS6KA3, PUM2, QKI, SMURF2, BPTF, NEDD1, SMARCAD1, SYNJ1, MATR3, FBXW2, HMBOX1, CDYL, CLASP2, VEZF1, DCAF7, CAB39, TEAD1, DDX3X, SERINC1, USP25, GABPA, C16orf72, KLF12, NBR1, KRAS, ICK,CEP350, OTUD4, KAT2B, PDPK1, PAPOLA, DCUN1D1, MPP5, DENND5A, DYRK2, UBQLN1,SEC23A, WDFY3, RANBP9, ATXN1, NCOA7, PHF6, FNDC3B, ASAP1, EVI5, HS2ST1, ATP2A2, NIN, BRMS1L, THAP1, FEZ2, PRKACB, RECK, MSL2, KIAA0355, YTHDF3, UBE2W, LAMC1, NUFIP2, ZMYM4, ARID2, CCNYL1, FAM8A1, YWHAB, HNRNPH2, CNOT6, MSN, PTPN13, RHOT1, ZEB1, BMI1, PLS3, ARHGAP20, CLIP1, PPP2R5C, FERMT2 ITPR1, MEX3C, ETS1, HNRNPK, EPS8, TRIM33, LEPR, NR3C1, MBLAC2, MAP4K3,EIF2S1, PSIP1, CNOT4, AGFG1, XIAP, CRKL, CLASP1, PLCL1, ANKRD28, ZC3H15, CDK17, NRIP1, FXR1, CNN3, CNOT7, ELF2, AP1S2, BAZ2B, TBK1, CALU, RPS6KB1, OSBPL11, EGLN1, ZEB2, OXR1, ARID4B, AEBP2, PDIK1L, PM20D2, RLF, NDST1, PDCD10, CSNK1G3, SCAMP1, PCNP, HNRNPU, NAB1, ZNF217, SLC38A2, SEMA6D, DLC1, SLC23A2, RPS6KA2, JAZF1, EFNB2, CLIC4, GNAI3, GDI2, MTUS1, TRIM2, RHOA, PPM1B, MAP4K4, RGL1, RAB21, BRWD1, NFYA, KLF10, MBOAT2, TSC22D2, REV1, KLF9, KHDRBS1,ACACA, SH3PXD2A, PALM2-AKAP2, FMR1, ZFPM2, HECTD2, CFL2, MAP2 ,TIAL1, NR5A2, ETV5, PCMTD1, CITED2, SSH2, CCNJ, GOSR2, NIPA1, YWHAG, AMMECR1L, ZC3H6, AMBRA1, NRP2, MGAT2, ANK3, ZNF532, PHTF2, FLI1, LMO7, YPEL2, RAP2C, ACVR2A, PRDM1, NUDT4, FAM118B, VLDLR, SLC16A2, SPIRE1, PNRC2, GLS, ACVR2A, E2F5 ,BACH2, CTDSPL, SPRY4 |
