# Supplementary material for: Identification and Validation of PIK3CA as a Marker Associated with Prognosis and Immune Infiltration in Renal Clear Cell Carcinoma
Source: J Oncol. 2021 Jul 27;2021:3632576. doi: 10.1155/2021/3632576 (PMC8337125; doi:10.1155/2021/3632576)
Supplement: Supplementary Materials — Supplementary Figure 1: the distribution of PI3KCA expression in univariate and multivariable analysis. Supplementary Figure 2: correlation analysis of PIK3CA expression and the expression of STRN, C9orf102, REST, and NHLRC2 (LinkedOmics). Supplementary Figure 3. PPI network of MIR-200B, MIR-200C, and MIR-429 target networks (GeneMANIA). PPI network and functional analysis about the gene sets of MIR-200B, MIR-200C, and MIR-429 target networks. The different colors for the network nodes indicate the biological functions of the set of enrichment genes. Supplementary Table 1: the LeadingEdgeGene of MAPK1-kinase target network (LinkedOmics). Supplementary Table 2: the LeadingEdgeGene of MIR-302C target network (LinkedOmics). [file Supplementary Fig 3.pdf › 3632576.f1/Supplementary Fig 3.pdf]

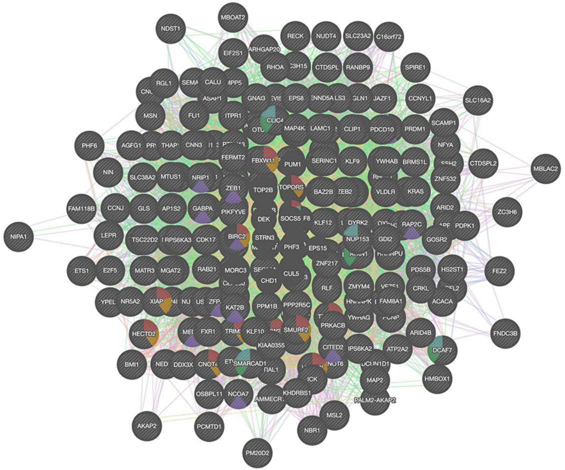

Networks

- Co-expression
- Physical Interactions
- Predicted
- Genetic Interactions
- Co-localization
- Shared protein domains
- Pathway

Functions

- small conjugating protein ligase activity
- acid-amino acid ligase activity
- transcription coactivator activity
- nuclear matrix
- nuclear periphery
